# BinaryCIF and CIFTools—Lightweight, efficient and extensible macromolecular data management

**DOI:** 10.1371/journal.pcbi.1008247

**Published:** 2020-10-19

**Authors:** David Sehnal, Sebastian Bittrich, Sameer Velankar, Jaroslav Koča, Radka Svobodová, Stephen K. Burley, Alexander S. Rose

**Affiliations:** 1 CEITEC, Central European Institute of Technology, Masaryk University, Brno, Czech Republic; 2 National Centre for Biomolecular Research, Faculty of Science, Masaryk University, Brno, Czech Republic; 3 Protein Data Bank in Europe (PDBe), European Molecular Biology Laboratory, European Bioinformatics Institute (EMBL-EBI), Wellcome Genome Campus, Hinxton, UK; 4 RCSB Protein Data Bank, San Diego Supercomputer Center University of California, San Diego, La Jolla, CA 92093, USA; 5 RCSB Protein Data Bank, Institute for Quantitative Biomedicine, Rutgers The State University of New Jersey, Piscataway, NJ 08854, USA; 6 Cancer Institute of New Jersey, Rutgers The State University of New Jersey, New Brunswick, NJ 08903, USA; 7 Skaggs School of Pharmacy and Pharmaceutical Sciences University of California, San Diego, La Jolla, CA 92093, USA; Hebrew University of Jerusalem, ISRAEL

## Abstract

3D macromolecular structural data is growing ever more complex and plentiful in the wake of substantive advances in experimental and computational structure determination methods including macromolecular crystallography, cryo-electron microscopy, and integrative methods. Efficient means of working with 3D macromolecular structural data for archiving, analyses, and visualization are central to facilitating interoperability and reusability in compliance with the FAIR Principles. We address two challenges posed by growth in data size and complexity. First, data size is reduced by bespoke compression techniques. Second, complexity is managed through improved software tooling and fully leveraging available data dictionary schemas. To this end, we introduce BinaryCIF, a serialization of Crystallographic Information File (CIF) format files that maintains full compatibility to related data schemas, such as PDBx/mmCIF, while reducing file sizes by more than a factor of two versus gzip compressed CIF files. Moreover, for the largest structures, BinaryCIF provides even better compression—factor ten and four versus CIF files and gzipped CIF files, respectively. Herein, we describe CIFTools, a set of libraries in Java and TypeScript for generic and typed handling of CIF and BinaryCIF files. Together, BinaryCIF and CIFTools enable lightweight, efficient, and extensible handling of 3D macromolecular structural data.

This is a *PLOS Computational Biology* Software paper.

## Introduction

Structural biologists are routinely using macromolecular crystallography (MX) and three-dimensional (3D) electron microscopy (3DEM) to produce atomic-level structural models (hereafter structures) of large biomolecular machines and depositing them to the single global archive of macromolecular structure data, known as the Protein Data Bank (PDB) [1]. Even larger and more complex 3D structures, such as the Nuclear Pore Complex (PDBDEV_00000012 [2]), are now coming from integrative (or hybrid) methods (IM) [3] that use multiple, complementary experimental and computational methods. These evolving and emerging structure determination methods require many new data items to (i) describe the state of the macromolecular system, (ii) to reflect the provenance, complexity, and quality of the underlying experimental data, (iii) to enumerate the computational procedure(s) used for 3D structure modeling, and (iv) to provide assessments of the validity of the structural model versus chemical reference and experimental data. With respect to file formats and data exchange in general the growth of structural biology poses two challenges. First, larger structural models require larger coordinate files and larger experimental data packages (e.g., ribosomes or multiple models obtained from time-resolved serial femtosecond crystallography). Second, larger molecular assemblies that are not resolvable at the atomic level require new multi-scale descriptions (e.g., coarse-grained beads representing single amino acid residues or irregular polygons representing protein domains or entire polypeptide chains).

The PDBx/mmCIF dictionary is an extensible data schema that describes macromolecular complex structures and associated metadata in exquisite detail. It is maintained by the Worldwide Protein Data Bank organization (wwPDB; wwpdb.org and [4]) in collaboration with scientific community working groups, including the PDBx/mmCIF Working Group (wwpdb.org/task/mmcif), the Hybrid/Integrative Methods Task Force [5], and the Small Angle Scattering Task Force [6]. PDBx/mmCIF was adopted as the PDB archival format by the wwPDB organization in 2014 [7]. Since mid-2019, PDBx/mmCIF format files have been mandatory for all new MX structure depositions to the PDB [8]. Together, the PDBx/mmCIF data dictionary [9] and the development version of the IM extension dictionary [10] provide complete description of the necessary data categories and items for describing the structure models and associated metadata in the PDB archive and the PDB-Dev [3] prototype system for IM structural models.

Data compression techniques are implemented for many different data types including text, images, video, audio, but also genome sequence, protein sequence, 3D atomic coordinates, etc to address the challenge of efficiently transmitting large sets of data over the internet so that they are easily accessible for anyone with a web-browser. To compress CIF formated macromolecular data, we will build on our earlier experience with the MacroMolecular Transmission Format (MMTF), which employed both lossy and lossless encoding strategies to reduce data redundancy and dynamic range (entropy) [11, 12]. Universal adoption of the MMTF format was never realized because it relied on a narrowly defined, non-standard data schema for describing macromolecular structures. Our approach combines MMTF encoding strategies with the global standard PDBx/mmCIF schema to create a compressed and extensible format. Instead of creating yet another file format, our approach involves compressible serialization of CIF data. Previously developed serialization formats for PDB data have not used CIF (e.g., PDBx/PDBML [13] support serialization of PDB structure data into XML by defining correspondences between the PDBx/mmCIF dictionary and an XML schema). A JSON based serialization format, PDBx/mmJSON [14, 15] (pdbj.org/help/mmjson) has also been proposed but does not include bespoke compression.

CIF is a lightweight, portable, and extensible format akin to JSON or CSV. Given appropriate software tooling CIF can be used to conveniently store and access macromolecular information, including data items that are not defined in the PDBx/mmCIF dictionary. To enable this use case and to tackle both the size and the complexity challenges posed by advances in structural biology, we created BinaryCIF, a binary compressed serialization scheme for CIF files, and CIFTools, a set of libraries in multiple programming languages (currently Java and TypeScript/JavaScript) for generic and typed access of CIF files.

## Design and implementation

CIF Overview: The syntax used in CIF data files and dictionaries is derived from the STAR (Self-defining Text Archive and Retrieval) grammar [16] and it has been revised to version 1.1. In its simplest form, a CIF file looks like a paired collection of data item names and values, where values can be numbers, text, lists, vectors, or tables. CIF uses data blocks to organize related information and data. Each data block contains one or more categories. Each category contains one or more columns. Each column within a category contains the same number of rows. More formally, a CIF file can be described using the list of interfaces given in Code listing 1, where the undefined value represents the dot (.) CIF token, the unknown value is the question mark CIF token (?).

CIFTools: With the CIFTools library we created a simple non-opinionated interface to CIF data. We do not impose a specific data representation onto users of the library. Instead, we offer access to the data as it is laid out in the CIF files. Hence, the CIFTools expose a unified interface to access data in CIF and BinaryCIF files as specified in Code Listing Listing 1. This approach allows applications to have their own data structures optimized for particular use cases. Given a schema, the CIFTools library provides fully typed data access and also supports schema-less access to CIF files (e.g. for initial prototyping or when no schema is available). Automatic code generation is available to create schema code from CIF dictionary definition files. CIFTools have built-in support for schemas to represent mmCIF (including IM), CCD (Chemical Component Dictionary) and BIRD (Biologically Interesting Molecule Reference Dictionary) files provided by the wwPDB.

**Listing 1**. CIF/BinaryCIF file interfaces.

interface File {

 getBlock(index: number): Block

}

interface Block {

 header: string

 getTable(name: string): Table| undefined

}

interface Table {

 name: string

 rowCount: number

 getColumn(name: string): Column | undefined

}

interface Column {

 name: string

 getValue(rowIndex: number): Value

}

type Value = undefined | Unknown | Number | String

Given an instance of a CIF file, the interfaces described in Code Listing 1 can be used to access any value stored in the file. For example, to access the 3D position of the 10th atom inside a standard mmCIF format file that stores a molecular structure, we could use the code given in Code listing 2. Because both CIF and BinaryCIF files implement this interface, programs consuming the data can be oblivious to the source of the data. This approach means that adapting existing software that currently supports CIF format for use with BinaryCIF is very straightforward.

**Listing 2**. Access the 3D position of 10th atom inside a standard mmCIF file that stores a molecule.

block = file.getDataBlock(0)

atom_site = block.getCategory(’_atom_site’)

x = atom_site.getColumn(’Cartn_x’).getValue(9)

y = atom_site.getColumn(’Cartn_y’).getValue(9)

z = atom_site.getColumn(’Cartn_z’).getValue(9)

BinaryCIF: The BinaryCIF format abstracts the structure of CIF formatted data and serializing it in a different way than text-based CIF files. Specific differences between BinaryCIF and text-based CIF are that in BinaryCIF, data is stored binary-encoded and column-oriented. The column orientation results in similar information being grouped together. In a text-based CIF file a single line (i.e. a row) may provide information for a single atom in a molecule. Thus, each row is a conglomerate of identifying information, 3D coordinates and other items; all of different data types. BinaryCIF follows a column-centric orientation of this data and groups similar information together while retaining the overall hierarchy of the original CIF file. This approach permits creation of a single array of float values to describe a particular data item (e.g., the x-coordinates of all atoms). Consequently, this arrangement of data enables more efficient encoding versus standard means alone (such as gzip) and is also leveraged by PDBx/mmJSON [14] and MMTF [12].

Each column is compressed separately using a combination of one or more of the compression methods enumerated in Table 1; many encodings are identical to MMTF [12] with the addition of Interval Quantization and String Array encoding. By default, the BinaryCIF encoder as implemented in CIFTools will determine the combination and parameters of encodings (Table 1) that is optimal, resulting in the minimal number of bytes. Alternatively, the user can specify encoding for specific columns (e.g., for 3D coordinate data requiring Fixed Point encoding to convert float values to integers). For lossless encoding of the Cartn_x, Cartn_y and Cartn_z mmCIF categories, values are multiplied by 1,000. Smaller values can be specified for lossy encoding which reduces 3D coordinate precision realizing smaller file sizes. If present, the unknown (?) and undefined (.) values in a category are handled by a dedicated mask that is an integer array describing for each row if the corresponding value is present or absent. This array tends to be uniform for a large fraction of cases and can be readily encoded with the previously described strategies (e.g. Run Length encoding).

**Table 1:** Encodings types supported by the BinaryCIF format.

Encoding	How it works	Useful for
Byte Array	Directly store data	Raw data that does not benefit from any encoding
Fixed Point	Multiply numeric value by a constant and store it as an integer	Floating point values where precision can be reduced (i.e. coordinate data)
Run Length	Store repeating numeric elements as a tuple with the value and the number of repeats	When combined with delta encoding, useful for storing linear identifiers
Delta	Instead of storing absolute values, store differences between consecutive elements	Linear identifiers & when combined with fixed point and integer packing, coordinate data
Interval Quantization	Store an interval quantized into 256 (8-bit) or 65536 (16-bit) uniformly distributed discrete steps (values are rounded to the closest step)	Experimental (density) data
Integer Packing	Represent large values using 8 or 16-bit numbers	Sequences of data where most values are small
String Array	Store an array of strings by concatenating all unique strings as pairs of substring indices into the concatenated one. Effectively encodes repeating substrings.	All string data, particularly annotations such as residue names

In summary, BinaryCIF is a map-like hierarchy that contains all information about the employed encodings in a self-descriptive manner. This representation can be readily encoded using MessagePack (msgpack.org). The resulting BinaryCIF file is self-descriptive vis-a-vis the combination and parameters of encoding steps used.

String Array Encoding: String data in columns such as atom names or amino acid labels tends to be highly repetitive. Such redundancy is addressed by a dedicated String Array encoding which concatenates all unique occurrences of a string 1. An array of integer offsets describes the positions where the original strings exist as substrings in the data string. Another array of indices captures the order in which these substrings occurred in the original column. Offsets and index arrays are encoded optimally by managing them as normal integer arrays.

Interval Quantization: Interval Quantization encoding is used to handle experimental 3D electron density maps from MX or electric coulomb potential maps from 3DEM. It is lossy by design, representing float values in an interval between minimum (min) and maximum (max) values using a defined number of equally-sized bins. The lossy encoding stores the index of the bin that optimally represents a value. Either 256 or 65,536 bins are used as these values capture the maximal information in 1 or 2 bytes, respectively. A similar strategy can be used to omit outliers if min and max values are chosen accordingly.

**Fig 1:**
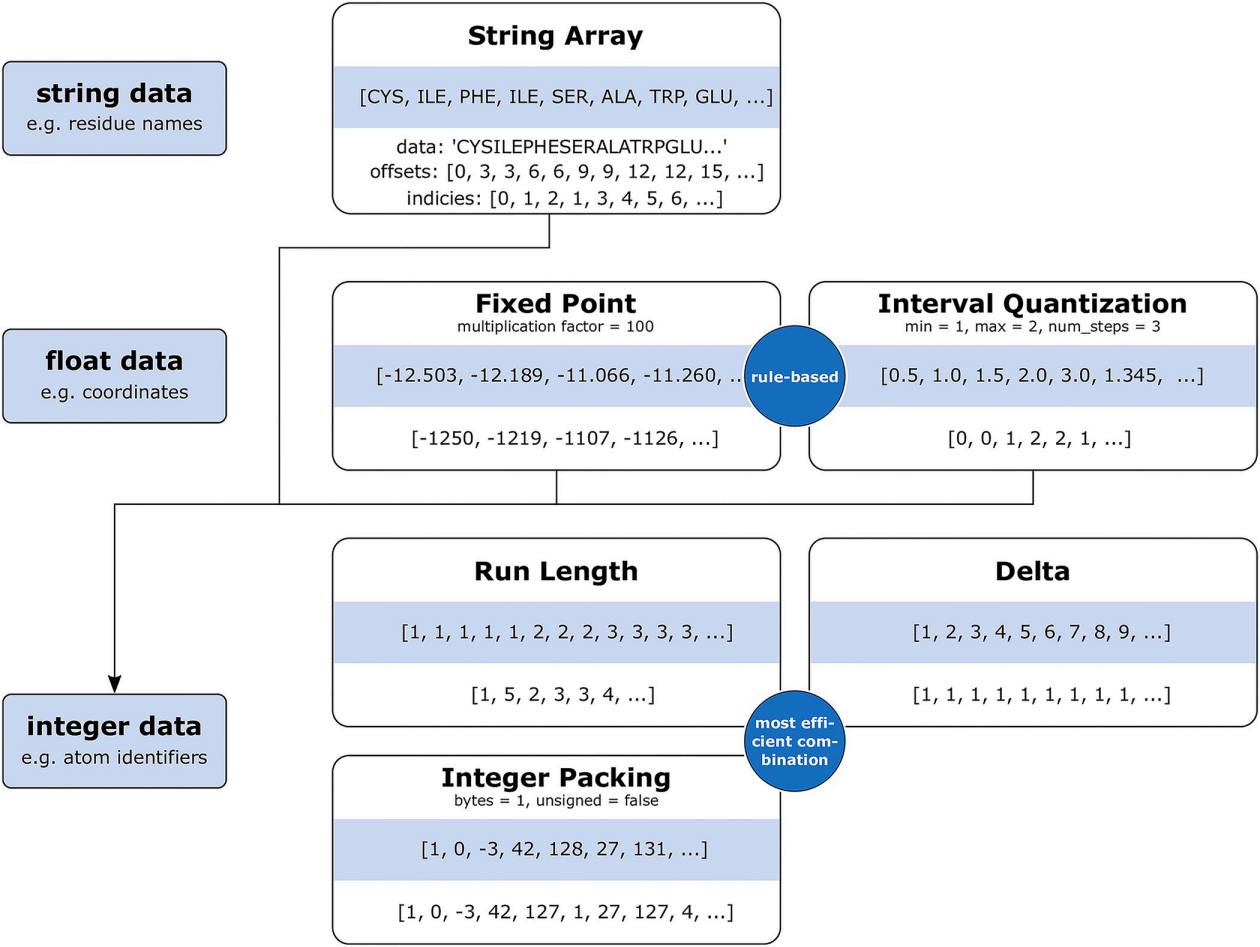
Compression strategies of BinaryCIF. The BinaryCIF codec represents diverse data types in a standardized manner: The indices wherein particular strings occur together with float values can be encoded as integer values. Interval Quantization is the only lossy encoding. For integer arrays the most efficient combination of Run Length, Delta, and Integer Packing is detected. This approach allows management of arbitrary data and even columns that are not defined by any schema. MessagePack is employed downstream of BinaryCIF encoding.

Established Encodings: Fig 1 illustrates encoding strategies employed in BinaryCIF. For implementation details of previously developed encodings see earlier LiteMol [17] and MMTF [11, 12] publications. Fixed Point encoding allows lossless representation of float values with n decimal places by multiplying each value with 10*^n^*. Delta encoding stores only the change of a numeric value with respect to its successor and is, for example, suitable for encoding 3D coordinates of covalently bonded atoms [11]. Run Length encoding is suitable for repetitive values, such as residue numbers, and collapses sequential repetitions of an integer into a tuple of the value and the number of occurrences.

Combinations of these strategies enable efficient data encoding. For example, atom identifiers that enumerate each atom with a unique integer value. Their value increases by 1 with each row, thus Delta encoding yields a vector of 1s that can be collapsed by the Run Length codec to the value 1 and the number of repetitions equal to the number of atoms. BinaryCIF implementations in CIFTools employ classifiers that determine the most efficient combination and parameterization of encodings automatically. This classification scheme is generic, as it does not require knowledge of the kind of data in a given column and thus works seamlessly for arbitrary information added by a user or by future extensions of the PDBx/mmCIF schema.

## Results

We assessed the performance of the BinaryCIF format together with its Mol* (TypeScript) and Java library implementations in terms of archive size and read performance.

Archive Size: We obtained all mmCIF and MMTF files available from the PDB archive [4] on 18 July 2019 and converted the mmCIF files to BinaryCIF, yielding 154,015 PDB structure files in each format. BinaryCIF and mmCIF files were annotated with the chem_comp_bond category using Mol* [18] (this information is present in MMTF but not the mmCIF files). The MMTF schema retains only atomic coordinates and a small set of meta-information (see S1 Table); thus, pruned BinaryCIF and mmCIF files containing identical information were created.

### Compression performance

**Fig 2:**
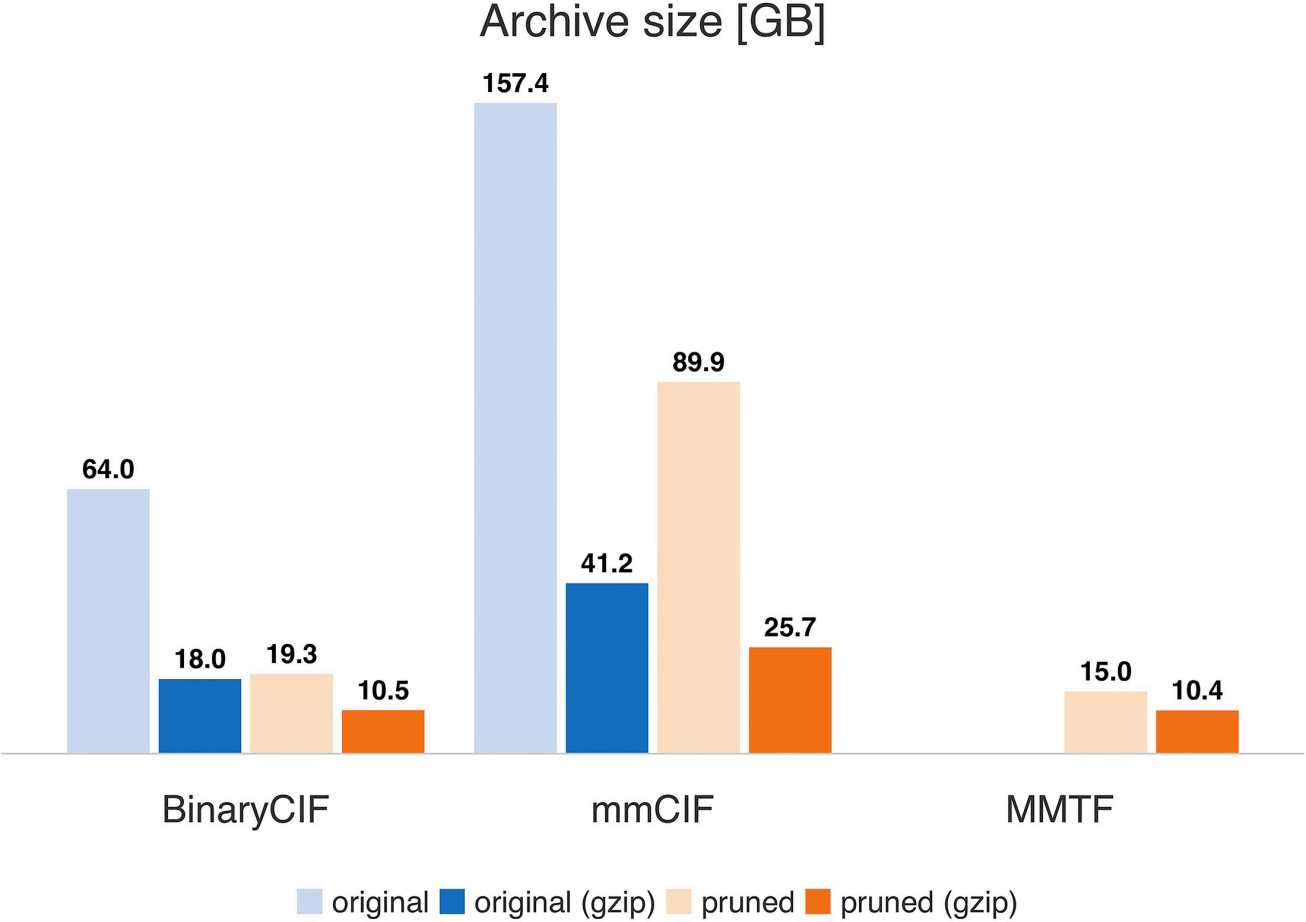
Archive sizes. Archive sizes for 154,015 files are given in GB (see S1 Text). Original refers to the content of the original structure files. Pruned resembles the set of information provided by MMTF files (see S1 Table). Use of BinaryCIF yields an archive size similar to MMTF.

Fig 2 depicts the archive size in various formats, either in their original state or providing only pruned information, and either uncompressed or compressed by gzip with default parameters. Pruning roughly halves the archive size when applicable. The pruned, gzipped BinaryCIF archive occupies 10.5 GB (marginally larger than the MMTF archive at 10.4 GB). When all data is present in the BinaryCIF representation (something not possible using MMTF), the compressed archive size is quite reasonable at 18.0 GB. As expected, binary formats store macromolecular data with high efficiency. In all cases, gzipping individual archive files drastically reduces their size; the effect is particularly pronounced for the text-based mmCIF format. The original version of the archive in BinaryCIF greatly benefits from gzip compression because the employed encoding strategies of each column are described as quite verbose strings (e.g. StringArray) that can be compressed efficiently.

**Fig 3:**
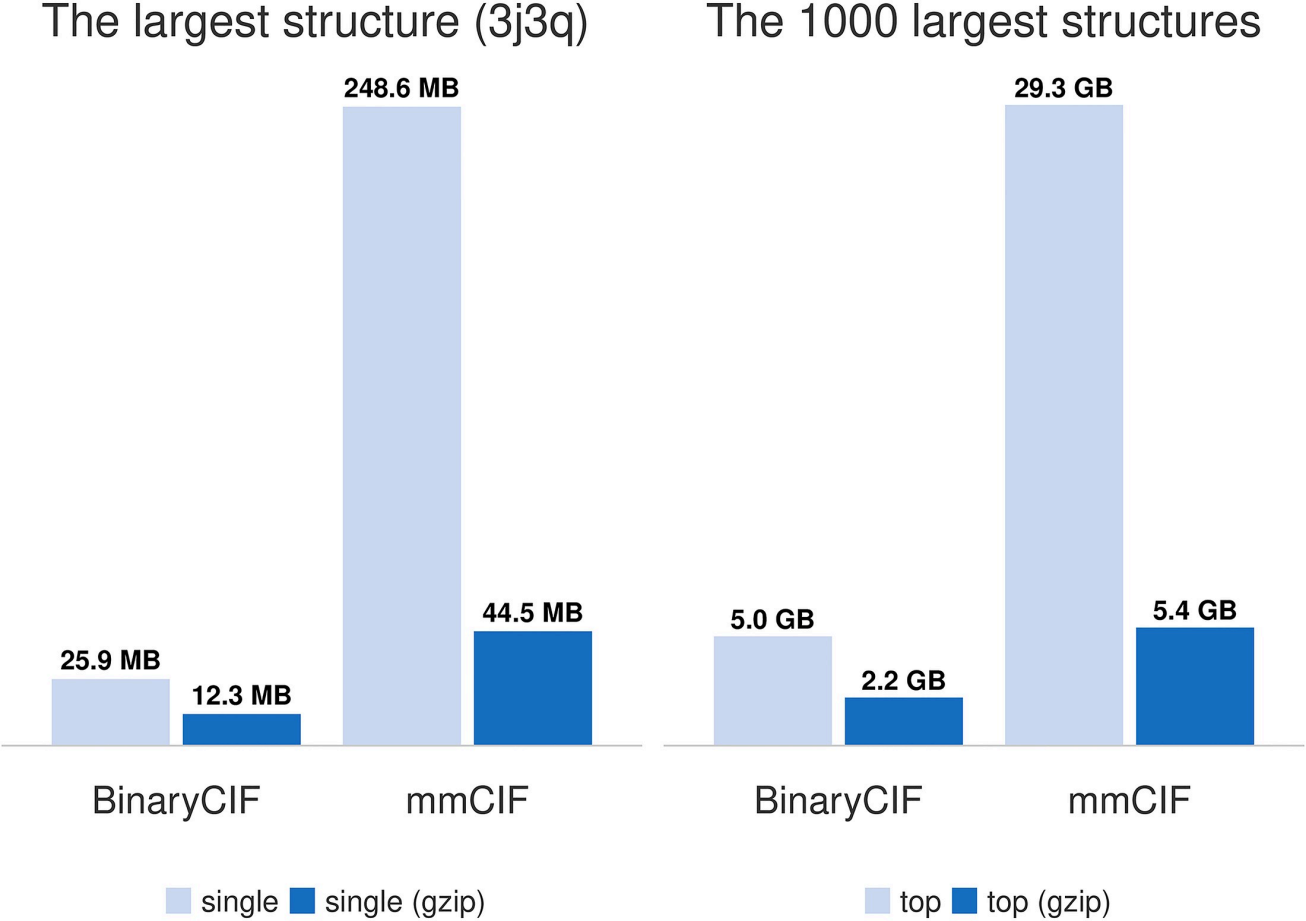
Large structures. BinaryCIF provides the most effective compression for the largest structures, enumerated in S2 Table.

An important advantage of the BinaryCIF format is, that the larger the data is, the higher compression factor that can be achieved with BinaryCIF. It is ideal for delivery and processing of macromolecular data, because whereas the operation with small-and intermediate-sized structures is relatively easy, work with enormous structures remains challenging making file size reduction essential. We examined the efficiency of BinaryCIF and gzipped BinaryCIF on four challenging structural biology data sets: The first contains only one structure of the human immunodeficiency virus or HIV capsid (PDB ID 3j3q), the largest structure stored in the PDB archive. The second data set contains the 1000 thousand largest structures found in the PDB (S2 Table). Fig 3 summarizes achievable compression levels. For 3j3q, BinaryCIF provides approximately factor ten compression versus CIF files and approximately factor four versus gzipped CIF files. For the 1000 largest PDB structures, BinaryCIF files are about six times smaller than CIF files, and gzipped BinaryCIF files are about 2.5 times smaller than gzipped CIF files. The third data set consists of 137,543 structure factor files that include all reflections used during x-ray structure determination (S1 Fig). Structure factors stored as BinaryCIF are about three times smaller than CIF files, gzipped BinaryCIF files provide about 1.5 times smaller file sizes than gzipped CIF files. The fourth data set contains the 1000 largest structure factor files (S2 Fig) and shows similar compression rates.

### Read performance

**Fig 4:**
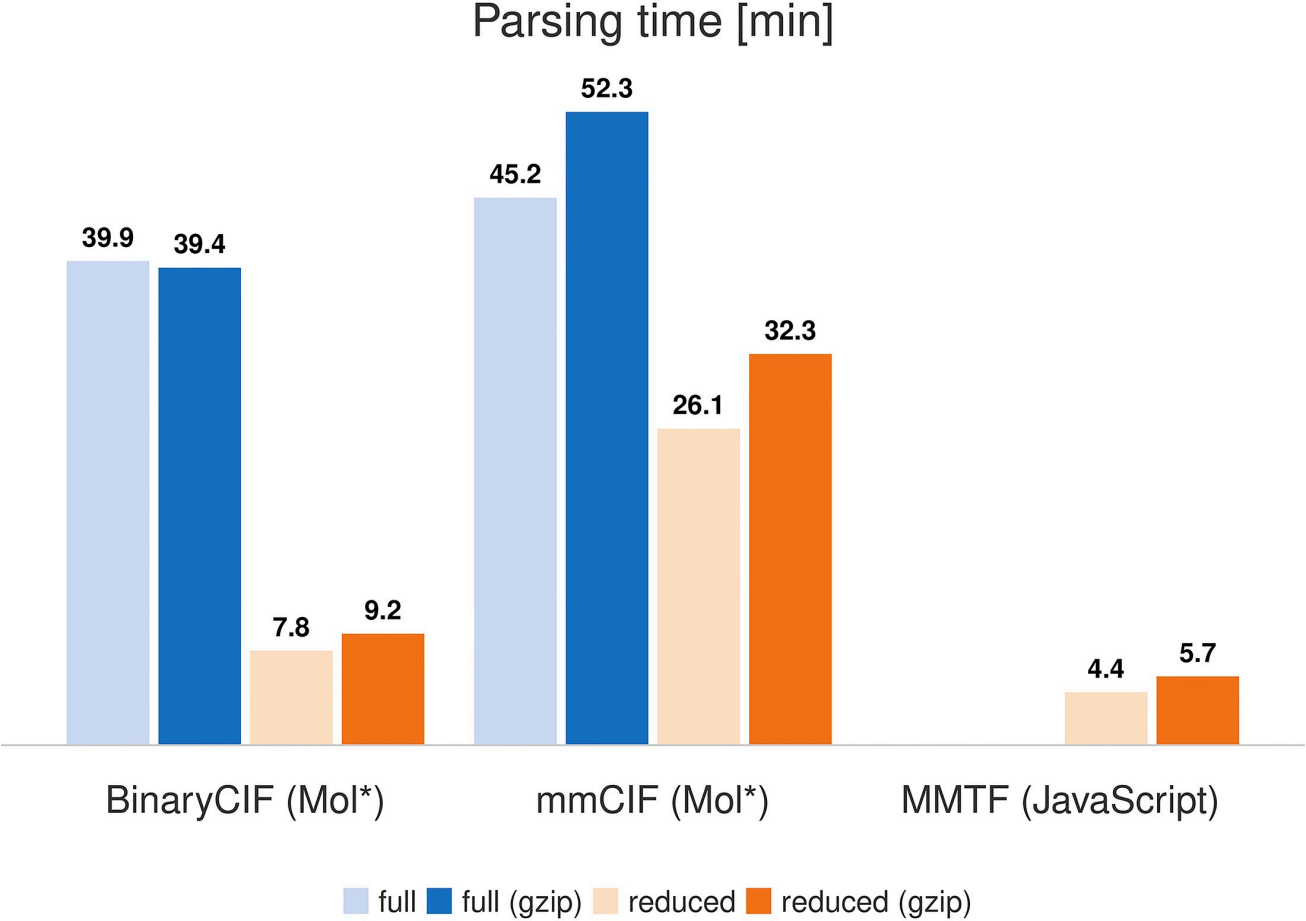
Read performance of JavaScript implementation. Average single-threaded parsing time for 154,015 PDB structures is given in minutes. Reading of binary data (BinaryCIF and MMTF) can provide a dramatic speedup. Handling gzipped files slows down parsing in most cases. Read performance can be easily improved by omitting less used meta-information as seen for the pruned bins.

**Fig 5:**
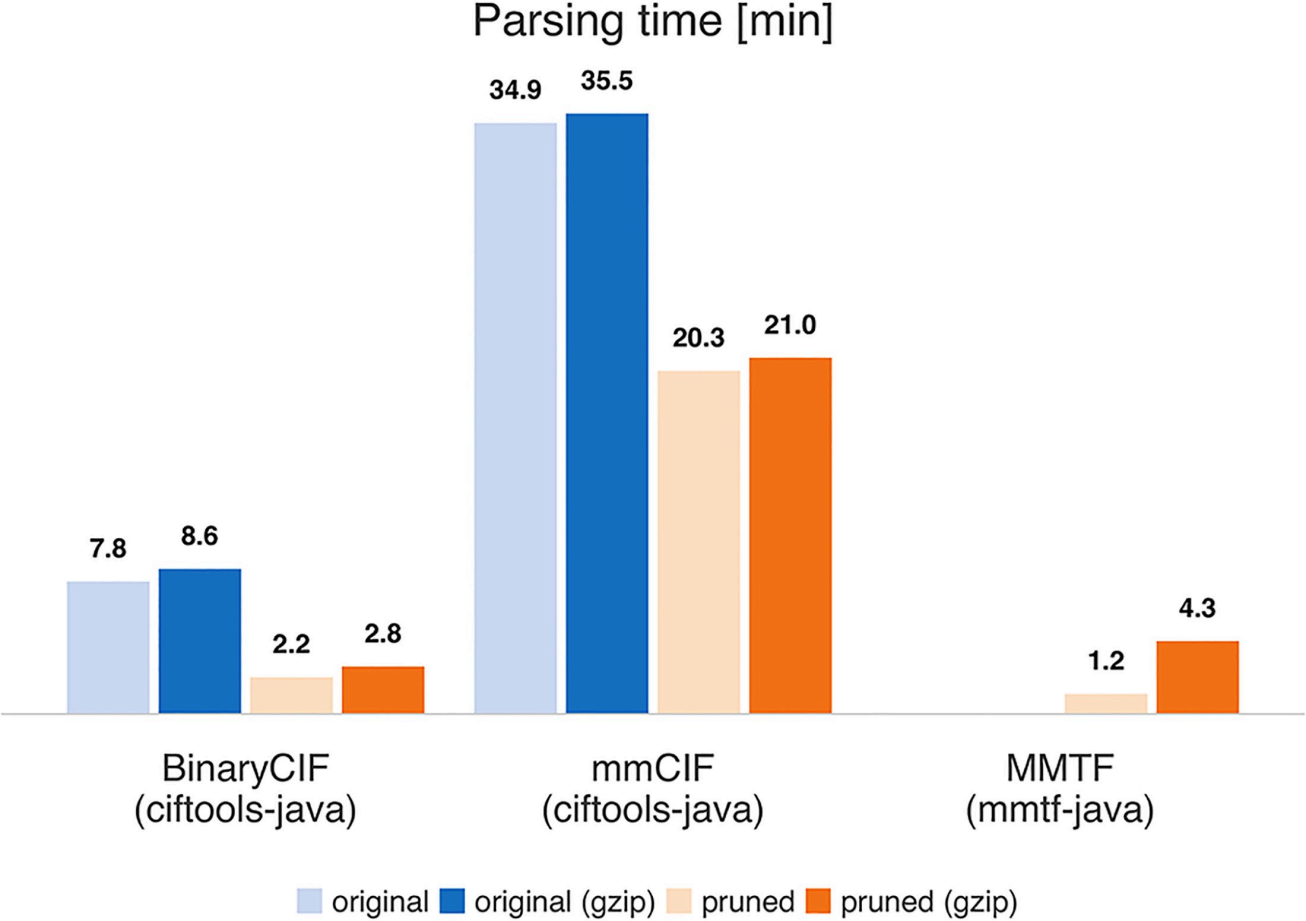
Read performance of Java implementation. Average single-threaded parsing time for 154,015 PDB structures are given in minutes.

After documenting that BinaryCIF enables efficient storage of macromolecular data, we assessed read performance for the entire PDB archive (see S1 Text). Average run times are provided in Figs 4 and 5. Again, we used the pruned representation of the archive for objective comparison. Pruning of information speeds up mmCIF and BinaryCIF parsing dramatically. Efficient data delivery, therefore, can be accomplished by omitting information from files that will not be required for the task at hand. For performance critical applications, such as searching the entire archive, custom versions of the archive can omit data for which access is not required. Creating such an archive is relatively straightforward using CIFTools (and the BinaryCIF/mmCIF specification).

Gzip compression leads to a decrease in read performance for all runs with exception of Mol* reading original BinaryCIF data. Potentially gzipping increases read performance on conventional HDDs where IO is slower. For our setup using a SSD, the trade-off between fewer IO operations and additional decompression CPU load is unfavorable.

mmCIF reading is slower due to larger data files and the need to parse the data. Read performance of binary data is higher: additional decoding steps are required but the amount of disk access is minimized and string parsing is avoided.

### Availability and future directions

CIFTools: We created open source CIFTools implementations in two languages, TypeScript and Java, and published them in common package repositories. Both implementations have support for reading and writing BinaryCIF and text-based CIF files plus CIF dictionary/schema management (for usage examples see S2 Text). The TypeScript implementation was developed on GitHub as part of Mol* (github.com/molstar/molstar), is made available as a package on NPM (npmjs.com/package/molstar) and archived as 10.5281/zenodo.3947316. The Java implementation is also developed on GitHub (github.com/rcsb/ciftools-java), available as a package on Maven (search.maven.org/artifact/org.rcsb/ciftools-java/) and archived as 10.5281/zenodo.3948501.

BinaryCIF: The new format is stable at Version 1.0 and ready to be implemented by interested parties. The full BinaryCIF specification is freely available on GitHub (github.com/molstar/BinaryCIF) and archived as 10.5281/zenodo.3947470. BinaryCIF was originally developed for use in LiteMol [17] including the CoordinateServer and DensityServer which have been in production use at PDBe since 2017. The use of BinaryCIF for volumetric data provides significant advantage over using the CCP4/MRC format by providing the ability to deliver multiple volumes in a single request, including additional metadata, at negligible computational overhead (see supplementary information of [17]).

A number of software applications and libraries already include support for handling BinaryCIF data. The Mol* Viewer (molstar.org/viewer, [18]) implements full BinaryCIF support and uses it for data delivery in its ModelServer and VolumeServer data delivery tools and to load PDBx/mmCIF files encoded as BinaryCIF. The BioJava project (biojava.org) has support for reading and writing macromolecular model BinaryCIF files using the ciftools-java package (github.com/rcsb/ciftools-java). Jmol (github.com/BobHanson/Jmol-SwingJS) has support for reading and writing electron density maps as BinaryCIF. The python-ihm package (github.com/ihmwg/python-ihm) supports reading and writing BinaryCIF files containing IM mmCIF data.

### Discussion

Herein, we presented tools for lightweight, efficient and extensible handling of 3D macromolecular structure data of ever-growing size and complexity. Our BinaryCIF serialization format provides state-of-the-art compression, while maintaining full compatibility with current and future CIF data schemas. For example, BinaryCIF is compatible with the PDBx/mmCIF format required for all MX depositions to the PDB archive. It is also already compatible with the emerging CIF schema for 3D structural models obtained from integrative methods. We achieve compatibility by decoupling the ‘what-is-stored’ in a file from the ‘how-it-is-stored’. BinaryCIF always stores the same data as its corresponding CIF file but it does so more efficiently to support faster loading of smaller files. Performance can be further improved by creating CIF files tailored to specific tasks (e.g., atomic displacement parameters and deposition details are not needed to compute structure alignments). The pruned mmCIF files provided for comparison with MMTF illustrate this very well. Users have the flexibility to remove (or add) data as they see fit.

The CIFTools libraries (presently available for Java and TypeScript) provide an efficient mechanism way for developers to add support for reading and writing Binary and CIF files alike to their programs without imposing any specific data model for providing access to all the data categories and items in the file. With respect to the ever changing field or structural biology it is important for file formats to keep up with changes necessary for describing and managing new data items while maintaining backwards compatibility. The mmCIF dictionary has proven readily extensible, with many new biologically crucial data categories added since its inception. The mmCIF schema is also versatile enabling automatic transformations into XML (PDBx/PDBML) and JSON (mmJSON). Our work adds transparent binary serialization and state-of-the-art compression to the CIF toolbox.

## Supporting information

S1 Fig Size of structure factors. Snapshot of 137,543 files as of 8 July 2020.
(PDF)

S2 Fig Size of largest structure factors. (PDF)

S1 Table The mmCIF categories used for the reduced representation. (PDF)

S2 Table The 1000 largest entries in the PDB archive. (XLSX)

S1 Text Benchmark details. (PDF)

S2 Text Usage examples. (PDF)
