# Supplementary figures and images for: BinaryCIF and CIFTools—Lightweight, efficient and extensible macromolecular data management
Source: PLoS Comput Biol. 2020 Oct 19;16(10):e1008247. doi: 10.1371/journal.pcbi.1008247 (PMC7595629; doi:10.1371/journal.pcbi.1008247)

# Structure factor size [GB]

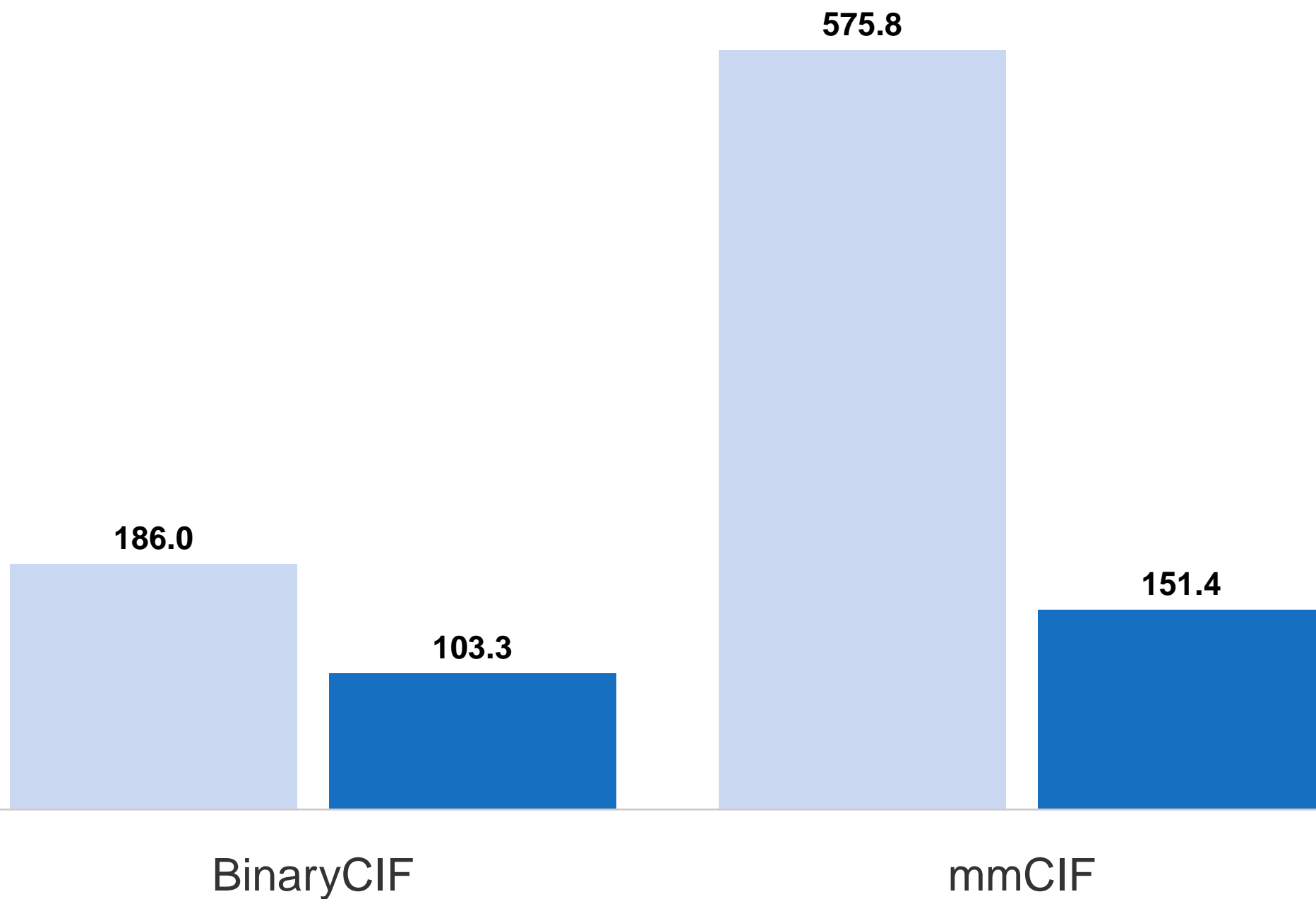

original original (gzip)

Supplement: S1 Fig — Snapshot of 137,543 files as of 8 July 2020. (PDF) [file pcbi.1008247.s001.pdf]

# The 1000 largest structure factor files [GB]

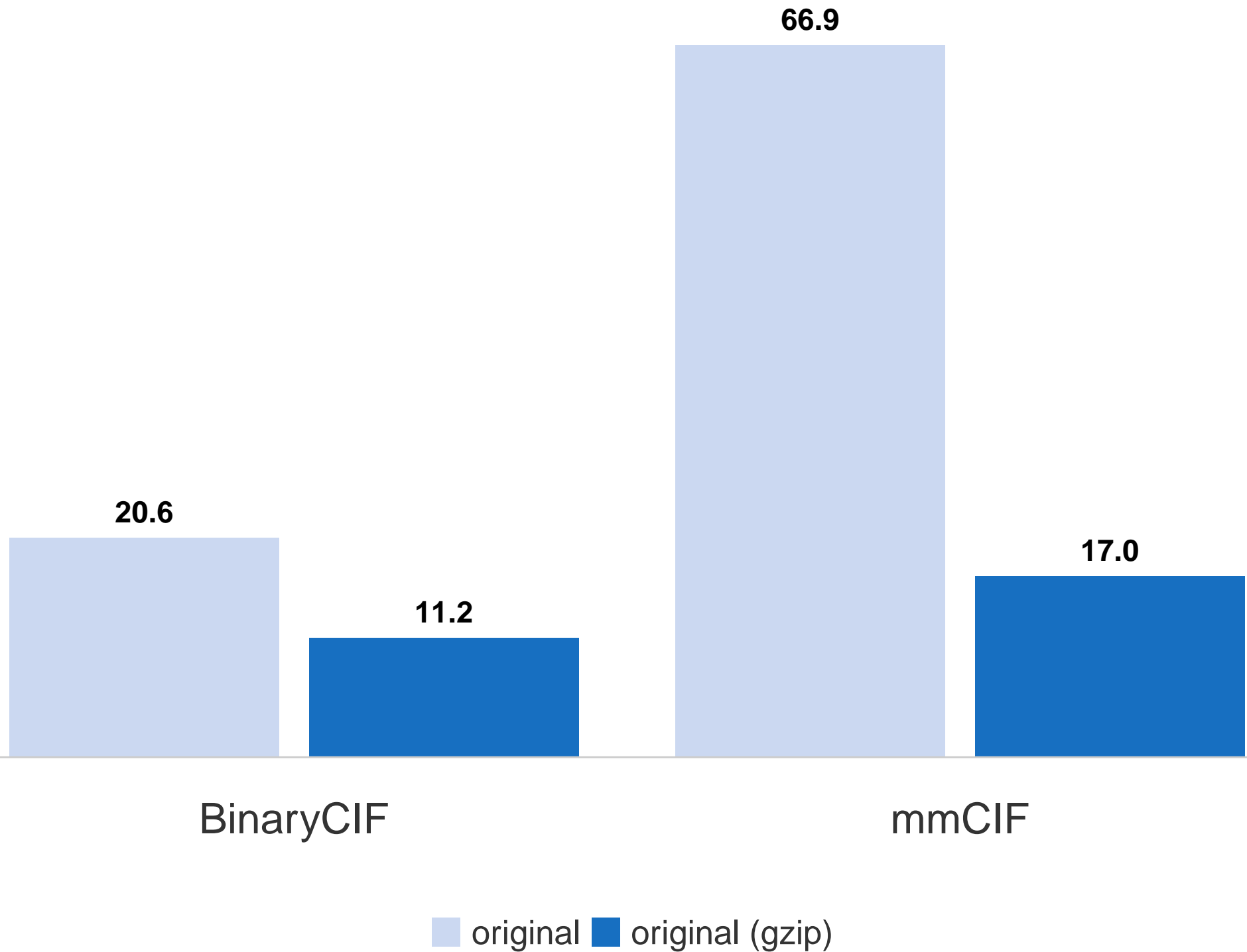

Supplement: S2 Fig — (PDF) [file pcbi.1008247.s002.pdf]
