# Supplementary material for: BinaryCIF and CIFTools—Lightweight, efficient and extensible macromolecular data management
Source: PLoS Comput Biol. 2020 Oct 19;16(10):e1008247. doi: 10.1371/journal.pcbi.1008247 (PMC7595629; doi:10.1371/journal.pcbi.1008247)
Supplement: S1 Table — (PDF) [file pcbi.1008247.s003.pdf]

Category name	Column name
atom_site	id
	type_symbol
	label_atom_id
	label_alt_id
	label_comp_id
	label_asym_id
	label_seq_id
	pdbx_PDB_ins_code
	Cartn_x
	Cartn_y
	Cartn_z
	occupancy
	B_iso_or_equiv
	pdbx_formal_charge
	auth_asym_id
cell	length_a
	length_b
	length_c
	angle_alpha
	angle_beta
	angle_gamma
chem_comp	id
	type
	name
chem_comp_bond	
entity	id
	type
	pdbx_description
entity_poly	entity_id
	pdbx_seq_one_letter_code
	pdbx_strand_id
entry	id
exptl	method
pdbx_audit_revision_history	revision_date
pdbx_database_status	recvd_initial_deposition_date
pdbx_struct_assembly_gen	
pdbx_struct_oper_list	
refine	ls_d_res_low
	ls_R_factor_R_free
	ls_R_factor_R_work
struct	title
struct_conf	conf_type_id
	id
	beg_label_asym_id
	beg_label_seq_id

	pdbx_beg_PDB_ins_code
	end_label_asym_id
	end_label_seq_id
	pdbx_end_PDB_ins_code
struct_conn	
struct_ncs_oper	
struct_sheet_range	id
	beg_label_asym_id
	beg_label_seq_id
	pdbx_beg_PDB_ins_code
	end_label_asym_id
	end_label_seq_id
	pdbx_end_PDB_ins_code
symmetry space_group_name_H-M	
