# Supplementary material for: BinaryCIF and CIFTools—Lightweight, efficient and extensible macromolecular data management
Source: PLoS Comput Biol. 2020 Oct 19;16(10):e1008247. doi: 10.1371/journal.pcbi.1008247 (PMC7595629; doi:10.1371/journal.pcbi.1008247)
Supplement: S1 Text — (PDF) [file pcbi.1008247.s005.pdf]

S1 Text

Benchmark Details

Measurements were made using a single core of a 3.2 GHz Intel Core i7 CPU and 16 GB memory, running the macOS as operating system. Reads were performed from SSD. Two warm-up iterations were performed, followed by 5 measurement iterations. Benchmarks of Java implementations were executed using JMH Java Benchmark Harness using Oracle JDK (HotSpot) 1.8.0 201-b09. Transpiled TypeScript was executed using Node v11.10.1.

The archive sizes reported refer to the total size of a single folder containing all 154,015 files. Values were obtained on macOS with APFS as the file system.
