# Supplementary material for: BinaryCIF and CIFTools—Lightweight, efficient and extensible macromolecular data management
Source: PLoS Comput Biol. 2020 Oct 19;16(10):e1008247. doi: 10.1371/journal.pcbi.1008247 (PMC7595629; doi:10.1371/journal.pcbi.1008247)
Supplement: S2 Text — (PDF) [file pcbi.1008247.s006.pdf]

Usage Examples

Typescript/Mol*

This requires node.js (https://nodejs.org/) to be installed. In a terminal, issue `npm install -g molstar` to install the molstar package.

Then, use `cif2bcif file.cif file.bcif` to convert any cif file to a bcif file. See `cif2bcif -h` for more options. To generate code from cif dictionaries see help of the `cifschema -h` command. For instance `cifschema --preset mmCIF` to generate TypeScript types describing mmCIF files for use with Mol*.

Java

This requires a Java Runtime Environment (https://www.java.com/en/download/) to be installed and the ciftools-java dependency to be imported e.g. using Maven:

```
<dependency>
    <groupId>org.rcsb</groupId>
    <artifactId>ciftools-java</artifactId>
</dependency>
```

Then, any PDB identifier can be downloaded in cif format from RCSB PDB and the corresponding bcif file will be written to the specified path.

```
import org.rcsb.cif.CifIO;
import org.rcsb.cif.schema.StandardSchemata;
import org.rcsb.cif.schema.mm.MmCifFile;

import java.io.IOException;
import java.nio.file.Paths;

public class ConvertExample {
    public static void main(String[] args) throws IOException {
        MmCifFile cifFile = CifIO.readById("1acj").as(StandardSchemata.MMCIF);
        CifIO.writeBinary(cifFile, Paths.get("1acj.bcif"));
    }
}
```
